# An Exploration of Access to Tobacco Cessation Services Through Pharmacies in a Medically Underserved Area in California: Accuracy of Telephone Information Regarding Pharmacist-Furnished NRT

**DOI:** 10.1177/21501319251403072

**Published:** 2025-12-08

**Authors:** Sara Schneider, Deanna M. Halliday, Arturo Durazo, Anna V. Song, Dorie E. Apollonio

**Affiliations:** 1 Nicotine and Cannabis Policy Center, Health Sciences Research Institute, University of California, Merced, Merced, CA, USA; 2 UCSF Cardiovascular Research Institute, Center for Tobacco Control Research and Education, University of California, San Francisco, CA, USA; 3 Department of Public Health, School of Social Sciences, Humanities and Arts, University of California, Merced, California, USA; 4 Department of Psychological Sciences, School of Social Sciences, Humanities and Arts, University of California, Merced, CA, USA; 5 School of Pharmacy, University of California, San Francisco, San Francisco, CA, USA

**Keywords:** pharmacies, tobacco use cessation, nicotine replacement therapy, primary health care, information seeking behavior, health services accessibility

## Abstract

**Background:**

Residents of California’s San Joaquin Valley (SJV) have limited access to physicians and high rates of tobacco use. To address this issue, the state permits pharmacists to independently prescribe nicotine replacement therapy (NRT). Rural residents seeking information about such services may depend on telephoning pharmacies; this case study sought to determine whether information received by phone accurately aligned with information relayed in person.

**Methods:**

We collected data on pharmacist prescribing in a rural, low-resource California region in 3 stages: (1) all corporate and independent pharmacies in 11 SJV counties were identified (N = 609), (2) all eligible pharmacies (n = 586) were telephoned to determine if they provided pharmacist-prescribed NRT, then (3) researchers visited all pharmacies in 2 counties (Merced and Madera, n = 49) and requested participation in a survey regarding NRT access.

**Results:**

No pharmacies in the sample area reported they could prescribe NRT when called; when visited in person, 10% of pharmacies (5/49) reported they could, demonstrating inaccuracies by telephone.

**Conclusions:**

Residents in medically-underserved, rural areas who seek information about health care by telephone may not receive accurate information compared to in-person communications, limiting their access to available, effective services in pharmacies. This includes pharmacist-prescribed NRT, which remains underutilized despite being legally authorized, reflecting broader health access challenges.

## Introduction

Within California, the San Joaquin Valley (SJV) is a predominantly rural, under-resourced region^1^ where residents have limited access to conventional primary care providers.^2^ Residents also experience high rates of tobacco product use compared to coastal regions of the state; this combination makes it difficult to access tobacco cessation treatment despite high need.^3-5^

For regions like the SJV that face insufficient access to primary care, residents often rely on local pharmacies for health services.^6^ Pharmacies can provide free health guidance, allow patients to visit without an appointment, and offer extended hours of operation (e.g., weekends and evenings).^7-9^ A 2022 study of 11.7 million insured individuals in the United States found that they visited community pharmacies almost twice as often as they visited physicians or other healthcare providers; moreover, 10% of the sample who sought care at pharmacies over the course of a year did not make any visits to other healthcare providers.^10^ Previous research has described this as a pharmacy “positive care law” in which access to care is greatest in areas of highest deprivation.^11,12^

Pharmacists are also able to counsel patients on the proper use of prescription medications, including nicotine replacement therapy (NRT) products (e.g., nasal sprays). When pharmacists pair access to NRT with counseling in community practice settings, tobacco abstinence rates double or triple compared to NRT use without counseling.^13-16^ California pharmacists gained the ability to independently prescribe, or “furnish,” NRT medications without a supervising physician in 2016,^6,17^ (p. 493) in an effort to increase public access to effective tobacco cessation treatment. Relative to purchasing over-the-counter (OTC) NRT products, pharmacist-furnished NRT provides patients with access to counseling, higher medication dosages, and additional cessation modalities, which collectively increase the likelihood of successfully quitting.^15,18-20^ Lower-income people who smoke are price sensitive and, as a result, may not purchase combination NRT therapy that is more likely to achieve abstinence without a prescription, given its higher cost.^21-23^

Although residents of rural regions have greater access to pharmacies than to other healthcare providers, many residents of the SJV, similar to rural regions around the world, nonetheless live comparatively far from local pharmacies (in regions described as “pharmacy deserts”).^24^ Telepharmacy, or the provision of pharmaceutical services offered over the phone, has been proposed as a bridge for services in rural areas, allowing residents to receive information in advance of committing to traveling long distances to access medications.^25,26^ Patients living in rural regions have expressed strong demand for such services, as well as demonstrating access to mobile phones that allow them to contact and be contacted by pharmacies.^27^

Although telepharmacy has the potential to increase access to medications and allow pharmacists to address the needs of populations spread across large geographic areas, operational difficulties may limit the development of these services.^25,26^ Efforts to increase access to care through telepharmacy can break down if residents do not receive accurate information over the phone (i.e., information that does not align with what is relayed in person) that allows them to access medications in their region,^26^ and exacerbate marginalized patients’ already-lower trust in providers due to past experiences with bias, and who are more likely to be physically unable to reach pharmacies and seek in-person care.^11^

Receiving accurate information by phone is critical to bridging gaps in access to medications for rural residents, who may rely on this information to justify traveling long distances. This study sought to assess the extent to which information provided over the phone regarding medication access, specifically for NRT, was consistent with actual services provided in a high-need rural area in California’s SJV.

## Methods

### Study Design

Data were collected in multiple stages to analyze the availability of pharmacist-furnished NRT and the accuracy of information relayed about its availability by phone calls versus in-person visits. This study received ethical approval from the Institutional Review Board at the University of California San Francisco (UCSF; IRB #21-35317) and was conducted in alignment with the ethical guidelines outlined in the Declaration of Helsinki.

### Setting and Participants

We collected data from all corporate and independent pharmacies within 11 counties of California’s SJV and Sierra Foothills (Calaveras, Fresno, Kern, Kings, Madera, Mariposa, Merced, San Joaquin, Stanislaus, Tulare, and Tuolumne; N = 609) using the California Department of Consumer Affairs Board of Pharmacy’s database. Pharmacies were initially excluded if they did not meet the inclusion criteria (n = 23; e.g., served a specialty population, had an unlisted number). A small portion of pharmacies were excluded in the second stage of data collection if they were failed to be contacted by phone (n = 10), and additional pharmacies were excluded in the third stage for miscellaneous reasons (e.g., we found that they were a private pharmacy, not a pharmacy, permanently closed; n = 12). In Stage Three of data collection, each participant provided written informed consent via electronic signature prior to study participation; all participants in this stage received a $25 incentive e-gift card.

### Sampling and Recruitment

Following initial exclusions in Stage One of data collection, in Stage Two, trained student intern researchers called the remaining 586 pharmacies by telephone who met the inclusion criteria to determine if pharmacists could furnish nicotine inhalers. Student intern researchers made up to 3 attempts between November and December 2023 to contact these pharmacies. In Stage Three, 6 non-student researchers (including authors AVS, DMH, and SS) visited all corporate and independent pharmacies within a subregion of the SJV (n = 49): Merced City (n = 11) in February 2024, and Merced County (excluding Merced City; n = 20) and Madera County (n = 18) in March 2024. These counties were chosen in part because (1) all pharmacies within these locations had reported in Stage One that they either did not furnish NRT or did not respond, (2) these locations are relatively geographically central within the SJV, falling between counties more southern counties like Fresno and more northern counties like San Joaquin.

### Data Collection Procedures

#### Stage One: Preliminary Data Collection

All corporate and independent pharmacies within 11 counties of California’s SJV and Sierra Foothills were identified.

#### Stage Two: Telephone Survey

Student intern researchers received an in-person oral training from senior authors AD and AVS, while senior authors DEA and DT attended and provided training virtually, on how to conduct phone calls with pharmacies in November 2023. The same day, student intern researchers began calling all pharmacies who met the inclusion criteria to determine if pharmacists could furnish nicotine inhalers, asking, “*I heard you can get a nicotine inhaler from a pharmacy without a prescription from your doctor. Can I do that at your pharmacy?*” This question was drawn from previously validated studies.^28,29^ Researchers recorded their responses in a master shared spreadsheet, where they reported the results of each call attempt (e.g., answered; no answer), who they spoke to, if the pharmacy reported furnishing, and additional notes. Researchers did not voluntarily provide their identities, but if probed, identified themselves as student intern researchers. There were no instances of multiple staff members providing responses. Nicotine inhalers were mentioned specifically to distinguish access to NRT through pharmacist prescribing from access to over-the-counter NRT.

#### Stage Three: In-Person Verification

**Figure 1.**
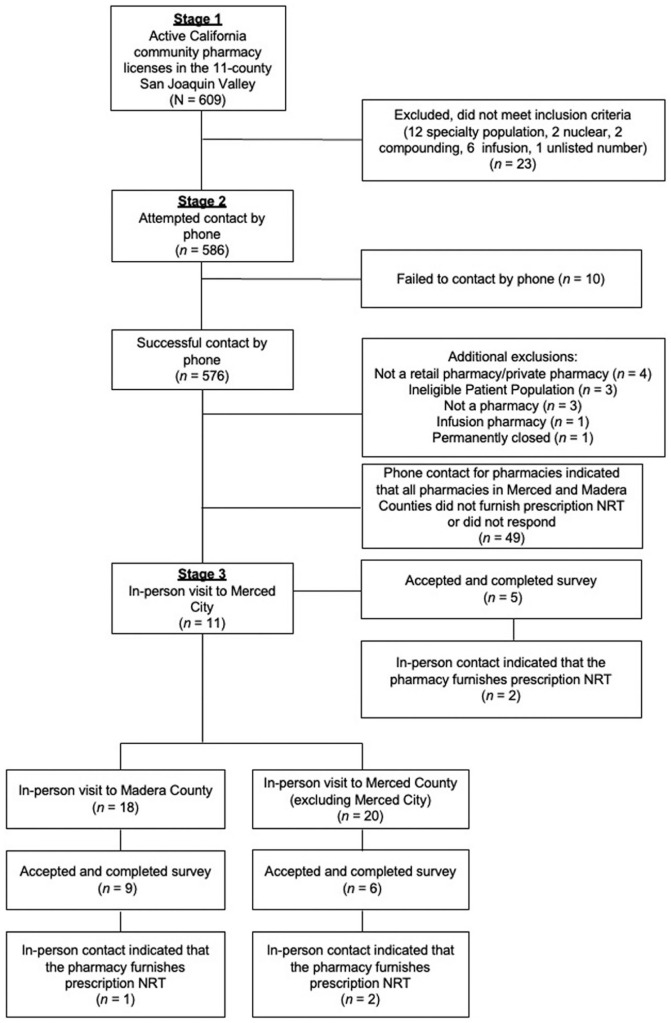
Flow chart of the data collection procedures, including inclusion and exclusion criteria.

To verify that our data collected by phone was accurately representative of in-person pharmacy furnishing practices, researchers visited all corporate and independent pharmacies within a subregion of the SJV: Merced City in February 2024, and Merced County (excluding Merced City) and Madera County in March 2024. Due to the discontinuation of nicotine inhalers in late 2023 due to a materials shortage,^30^ pharmacy personnel were asked, “*Can you furnish (prescribe) prescription nicotine replacement therapies like nasal sprays or gums at this pharmacy?*” See Figure 1 for a flow chart of the data collection procedures, including inclusion and exclusion criteria.

### Measures

The primary measure of interest was whether pharmacies reported furnishing NRT by phone versus in person. Additional notes were taken regarding call outcomes (e.g., no answer) and pharmacy staff responses.

### Data Analysis

Descriptive statistics were used to describe the accuracy or alignment of information relayed about pharmacist-furnished NRT by phone and in person. A Fisher’s exact test was conducted using R version 4.2.3 to analyze if there was a difference in the proportion of misreported furnishing practices between locations.

## Results

In the third stage of data collection, 5 (45%) of the 11 pharmacies within Merced City limits agreed to be surveyed. Among these 5, 3 (60%) were corporate pharmacies, and 2 (40%) reported that they furnished NRT; both of those that furnished were corporate pharmacies. Individuals who declined to be surveyed (n = 6) were asked if they were willing to answer 1 quick question, indicating whether they could furnish in their store. None of these individuals reported that they furnished in response to this 1-item question.

**Figure 2.**
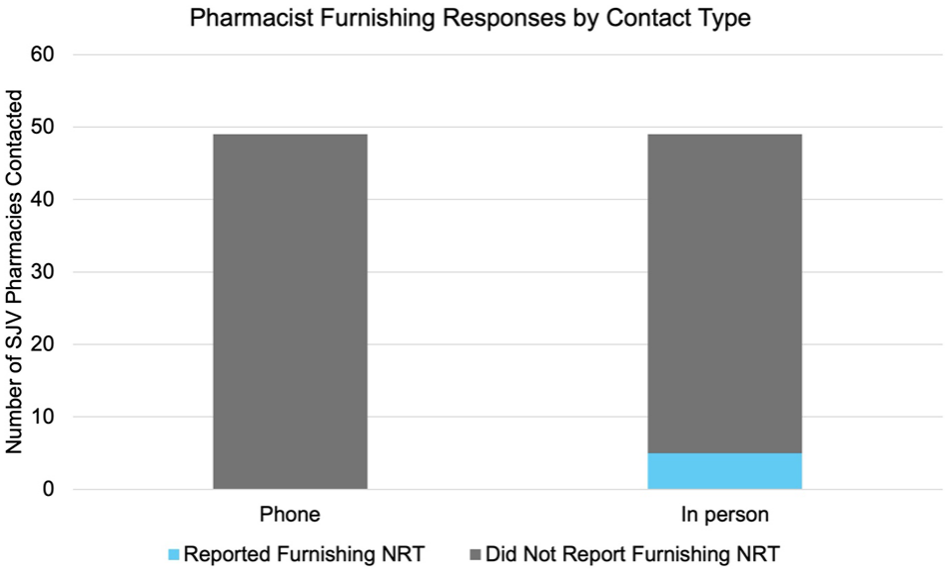
Bar chart illustrating variance in pharmacist furnishing responses by contact type (phone versus in person), across all locations (Merced City, Merced County, and Madera County).

Across Merced and Madera Counties (excluding Merced City), nearly 8% (3/38) of pharmacies reported furnishing NRT, and all were corporate pharmacies (Merced County: n = 2; Madera County: n = 1). Out of the 38 eligible pharmacies, 39% (15/38) agreed to be surveyed (Merced County: n = 9; Madera County: n = 6; corporate pharmacy: n = 8; independent pharmacy: n = 7). Individuals who declined to be surveyed (n = 23) were asked if they were willing to answer 1 quick question, indicating whether they could furnish in their store. Among those who declined to be surveyed, 4% reported that they did furnish (1/23; a Merced County corporate pharmacy), 70% (16/23) reported that they did not furnish, 4% did not know (n = 1), and all others declined to respond (n = 6). In total, 10% of pharmacies (5/49) across Merced City, Merced County, and Madera County indicated that they furnished NRT when they asked about this service in person, and all were corporate pharmacies (see Table 1; see Figure 2).

**Table 1.** Reported NRT Furnishing Availability by Contact Method and Subregion.

Region	Sample size (n (%))	Reported furnishing NRT by phone (n)	Reported furnishing NRT in person (n (%))	95% confidence interval
Madera County	18 (36)	0	1 (6)	[0.01,0.27]
Merced City	11 (22)	0	2 (18)	[0.02,0.52]
Merced County (excluding Merced City)	20 (41)	0	2 (10)	[0.01,0.32]
Total	49	0	5 (10)	[0.03,0.22]

Across Merced City, Merced County, and Madera County, 5 of these pharmacies with furnishing abilities were corporate pharmacies, and had all previously indicated that they did not furnish NRT when they were contacted by telephone. A Fisher’s exact test found no statistical difference in the proportion of misreported furnishing practices between locations (Madera County, Merced City, Merced County; *p* = .70; 3 missing responses and 1 “I don’t know” response from Madera County were omitted from the test).

## Discussion

Residents of rural areas increasingly attempt to access care through telepharmacy, which includes (but is not limited to) gathering information about the availability of medications. Previous research has detailed barriers to telepharmacy from the perspective of healthcare providers.^25,26^ Our findings expand on this work by identifying an additional barrier to access, namely that patients may not receive accurate or complete information from pharmacies about the services they provide. In our sample, 5, representing 10% of all pharmacies serving the area, reported they did not furnish NRT when contacted by telephone, but did in fact provide this service. As a result, residents of the region who attempted to contact pharmacies in advance would be unaware of tobacco cessation services available to them. This is particularly critical given that rural residents face long travel times in their efforts to secure care, even though pharmacies are more accessible than other healthcare providers.

Like residents of rural areas, researchers attempting to identify the extent to which pharmacies provide services also rely on phone contacts. Calling pharmacies to determine whether they prescribe medications has been a standard practice since at least 2018,^28^ reflecting the fact that response rates for studies that contact pharmacies by telephone are substantially higher than those relying on email or internet sampling.^31,32^ However, there have been limited efforts to assess whether information about medication access provided over the phone by pharmacies is accurate, although the COVID-19 pandemic has partially driven new efforts to research this topic. In 2023, Francis et al^33^ found high rates of overall accuracy when comparing patients obtaining a “best possible medication history” by telephone versus in person. For PrEP and PEP medications for HIV prevention, in 2024, Herron et al^34^ contacted 16 independent pharmacies in California’s San Francisco Bay Area by phone; they later determined, through in-person visits, that all pharmacies that had reported furnishing PREP and PEP medications when contacted by phone did in fact furnish these products.

Considering the high rates of accuracy for telecommunications in other settings, we speculate that this discrepancy in information relayed by phone versus in person may be due in part to pharmacy staff’s unfamiliarity with the practice of furnishing NRT as compared to other products like hormonal contraceptives. Specifically, recent findings demonstrate that SJV pharmacists do not perceive there to be a strong need or desire for these services,^35^ and relatedly, SJV residents have been found to be wary of taking NRT products due to concerns surrounding side effects and nicotine dependence.^36^ Thus, even if these services are available, they may not be utilized frequently by patients.

Given that pharmacy staff are the first trusted line of telecommunication for patients, proper staff training is needed to ensure patients receive accurate information without having to speak directly to the pharmacist. To encourage and educate the public, pharmacies can invest in relatively cost-efficient advertising of these services, such as physical placards, online promotions, and text messages, analogous to those often used for advertising vaccinations. Public directories of pharmacies who furnish NRT could also be created and advertised by pharmacies, public health departments, hospitals, etc.

This study has limitations. Due to the researchers’ limited available personnel and the distances between pharmacies in rural areas, it was impractical to attempt in-person visits for all pharmacies in the 11 counties we telephoned in the second stage of data collection, restricting researchers’ visits to a sample of all pharmacies within 2 counties. The cross-sectional nature of the in-person visits was also a limitation, where researchers were limited in the number of times they were able to visit pharmacies, and they may have visited during an inconvenient day or time. As such, these findings may limit generalizability beyond this geographic region, and to an extent within this region. The findings from this research also differed substantially from findings regarding pharmacist prescribing of HIV prevention medications in a 9-county, urban, coastal California region.^34^ There is insufficient information to assess whether these differences reflect characteristics of rural pharmacies relative to urban pharmacies, type of medication, independent versus corporate pharmacies, or other factors. Further research is needed to understand the potential influence of these factors, as well as to understand how residents living in pharmacy deserts and rural regions beyond the SJV and California gather information about available services.

## Conclusions

Pharmacies provide crucial access to health care in rural regions. In areas with comparatively high rates of tobacco use, governments have expanded the prescribing authority of pharmacists with the goal of increasing access to tobacco cessation services, particularly in areas with limited access to physicians. In our study of a largely rural, under-resourced area of California where residents have more access to pharmacy services than to conventional primary care, we found that although at least 10% of pharmacies could prescribe NRT and counsel patients on quitting, none of those pharmacies revealed that they did so when contacted by telephone. We speculate that the inaccurate information being relayed over the phone may lead residents who use tobacco products, and who are seeking assistance in quitting, to believe that they cannot easily access these services. Proper staff training is needed to ensure patients receive accurate information, and increased advertising and public directories of these services would increase patient awareness.
